# Genetic and Environmental Regulation on Longitudinal Change of Metabolic Phenotypes in Danish and Chinese Adult Twins

**DOI:** 10.1371/journal.pone.0148396

**Published:** 2016-02-10

**Authors:** Shuxia Li, Kirsten Ohm Kyvik, Zengchang Pang, Dongfeng Zhang, Haiping Duan, Qihua Tan, Jacob Hjelmborg, Torben Kruse, Christine Dalgård

**Affiliations:** 1 Unit of Human Genetics, Department of Clinical Research, University of Southern Denmark, Odense, Denmark; 2 Department of Clinical Research, University of Southern Denmark, and Odense Patient data Explorative Network (OPEN), Odense University Hospital, Odense, Denmark; 3 Qingdao Center for Disease Control and Prevention, Qingdao, China; 4 Department of Public Health, Qingdao University Medical College, Qingdao, China; 5 Epidemiology, Biostatistics and Biodemography, Department of Public Health, University of Southern Denmark, Odense, Denmark; 6 Environmental Medicine, Department of Public Health, University of Southern Denmark, Odense, Denmark; The University of Tokyo, JAPAN

## Abstract

**Objective:**

The rate of change in metabolic phenotypes can be highly indicative of metabolic disorders and disorder-related modifications. We analyzed data from longitudinal twin studies on multiple metabolic phenotypes in Danish and Chinese twins representing two populations of distinct ethnic, cultural, social-economic backgrounds and geographical environments.

**Materials and Methods:**

The study covered a relatively large sample of 502 pairs of Danish adult twins followed up for a long period of 12 years with a mean age at intake of 38 years (range: 18–65) and a total of 181 Chinese adult twin pairs traced for about 7 years with a mean baseline age of 39.5 years (range: 23–64). The classical twin models were fitted to the longitudinal change in each phenotype (Δphenotype) to estimate the genetic and environmental contributions to the variation in Δphenotype.

**Results:**

Moderate to high contributions by the unique environment were estimated for all phenotypes in both Danish (from 0.51 for low density lipoprotein cholesterol up to 0.72 for triglycerides) and Chinese (from 0.41 for triglycerides up to 0.73 for diastolic blood pressure) twins; low to moderate genetic components were estimated for long-term change in most of the phenotypes in Danish twins except for triglycerides and hip circumference. Compared with Danish twins, the Chinese twins tended to have higher genetic control over the longitudinal changes in lipids (except high density lipoprotein cholesterol) and glucose, higher unique environmental contribution to blood pressure but no genetic contribution to longitudinal change in body mass traits.

**Conclusion:**

Our results emphasize the major contribution of unique environment to the observed intra-individual variation in all metabolic phenotypes in both samples, and meanwhile reveal differential patterns of genetic and common environmental regulation on changes over time in metabolic phenotypes across the two samples.

## Introduction

Metabolic disorders including obesity, impaired glucose regulation, dyslipidemia, and hypertension are among the top preventable risk factors in association with the development of type 2 diabetes and atherosclerotic cardiovascular disease (CVD) [1–3]. Metabolic phenotypes e.g. blood glucose, blood lipids, blood pressure, and body mass index are, similar to most complex traits, regulated by both genetic and environmental factors with the interaction between them as central to the development of metabolic abnormality and diseases [4,5]. In the literature, the genetic and environmental contributions to metabolic phenotypes and metabolic diseases have been intensively studied using family [6–8] and twin [9–14] data with interesting results pointing to significant genetic and environmental regulations on the level of metabolic phenotypes.

Although the levels of metabolic traits are good indicators of an individual’s health status and provide the basis for defining and diagnosis of metabolic abnormality, the rate of change of metabolic phenotypes in adults may be more indicative of disorder-related modifications and disease onset [15] given the fact that metabolic profiles are age dependent [16,17]. This is true not only for metabolism but also for human health in general. For example, based on 10 years follow-up data, Turiano et al. [18] reported that longitudinal change in personality traits are associated with self-reported health outcomes. From a public health point of view, studying the individual progression of metabolic traits may contribute to personalized approaches in health care and for disease control. Likewise, dissecting the genetic and environmental regulation of the intra-individual change over time in metabolic traits can help with development of more effective strategies for intervention and prevention. Although the genetic and environmental influences on the level of metabolic phenotypes have been intensively studied using twin methods, twin studies on longitudinal change in metabolic phenotypes have been rare due to high expense, loss of follow up, and long waiting time in prospective investigations. Nevertheless, there have been several longitudinal twin studies on metabolic phenotypes [19–26]. However, these studies were either limited to body mass traits (weight, height and BMI) [19–23] or focused on phenotype stability or correlation over ages instead of longitudinal change in metabolic phenotypes [24–26].

Based on a Danish-Chinese collaboration on twin studies, we collected longitudinal data on multiple metabolic phenotypes in Danish and Chinese adult twins. The two samples of twins represent western (Danish) and eastern (Chinese) populations of distinct ethnic, cultural, socio-economic background, and geographical environment, providing unique data for twin modeling on longitudinal patterns of multiple metabolic phenotypes within and for comparison across the two samples.

## Materials and Methods

### The Danish cohort

The Danish cohort consisted of twins originally recruited from the nationwide, population-based Danish Twin Registry during 1997–2000 to examine the importance of genes, family environment and individual environment for the development of insulin resistance, abdominal obesity and cardiovascular risk factors, i.e. the GEMINAKAR study as described previously [10, 27–29]. This cohort was followed up during 2010 to 2012. At baseline (time 1), the exclusion criteria included known diabetes or cardiovascular disease, conditions making a progressive maximal bicycle test impossible, pregnancy, and breast feeding. The cohort consisted of 756 twin pairs (783 females, 729 males, among them, 309 monozygotic (MZ) and 447 dizygotic (DZ) twin pairs) who underwent an extensive full day clinical examination of a variety of phenotypes. The mean age of the participants at baseline was 38 years, range: 18–67 years. At follow-up (time 2), 1139 twins agreed to participate of which a total of 502 complete pairs (545 females, 459 males), hereof 226 monozygotic (MZ) pairs and 276 dizygotic (DZ) pairs were available. Mean age at follow-up was 50 years, range: 30–75 years.

Twin zygosity was determined using microsatellite markers. All participants gave their written informed consent to participate and the local scientific committee of the Region of Southern Denmark (baseline, S-VF-19970271; follow-up, S-20090065) and Danish Data protection Board (baseline, 1999-1200-441; follow-up, 2009-41-2990) approved the study protocol.

### The Chinese cohort

The Chinese twin samples were collected by the Qingdao Twin Registry at the Qingdao Center for Disease Control and Prevention (Qingdao CDC). At baseline, twins were recruited randomly through residence registry and the local disease control network of Qingdao CDC in 2006–2007. Twins were excluded from the study due to pregnancy, breast feeding, known diabetes and/or cardiovascular disease and use of weight-reducing medicaments within one month [12,13]. Only complete twin pairs who participated both investigations at baseline (time 1) and follow-up (time 2) were included. The same procedure for data collection was applied at both baseline and follow-up studies. A total of 181 twin pairs (101 MZ and 80 DZ twin pairs) were identified with longitudinal measurements taken about 7 years apart with a mean age at baseline 39.5 (range: 23–64) years and at follow up 46.5 (range: 30–71) years. Among them 245 were females and 117 were males. Twin zygosity was determined by DNA testing using 16 short tandem repeat DNA markers at the central laboratory of Qingdao Blood Bank. The Chinese study was approved by the local ethics committee at Qingdao CDC, Qingdao, China.

### Phenotypes studied

Both studies covered 12 metabolic phenotypes, i.e. total cholesterol (TC), triglycerides (TG), high density lipoprotein cholesterol (HDL), low density lipoprotein cholesterol (LDL), fasting blood glucose (GLU), body weight (WT), body mass index (BMI), waist (WAIST), hip (HIP) circumference, waist-hip ratio (WHR), systolic (SBP) and diastolic (DBP) blood pressure. BMI was calculated as weight (kilogram, kg) divided by the square of height (meter, m) with body weight measured using a standing beam scale and to the nearest 0.1 kg and height measured using a vertical scale with a horizontal moving headboard and to the nearest centimeter. Waist and hip circumferences (in centimeter, cm) were taken in standing position with waist circumference measured midway between the lowest rib and the iliac crest, and hip circumference measured over the widest part of the gluteal region [12, 28]. Systolic and diastolic blood pressure measurements (mmHg) were taken after at least 5 minutes of rest following a standard procedure using a conventional mercurial sphygmomanometer. The mean of three measurements (taken at least 1 minute apart) was calculated and used in subsequent analyses. Blood glucose concentration (mmol/l) was analyzed by the glucose dehydrogenase oxidation method [12, 27] both for Danish and Chinese blood samples. TG, TC, HDL and LDL were measured (mmol/l) using standard clinical biochemical methods both for Danish twins (except LDL which was calculated from TC, HDL and TG using Friedewald formula) [29] and for Chinese twins [12].

### Statistical analysis and twin modeling

Statistical significance of longitudinal change for each phenotype was assessed by fitting the mixed effect kinship model [30–31] as *y = β*_*0*_*+β*_*1*_*age+ β*_*2*_*sex+ β*_*3*_*time+random effects*, where *y* stands for the phenotype values, with fixed effects for time (0 for baseline or time 1, 1 for time 2), baseline age at intake and sex, and random effect for twin pairing to account for the intra-pair genetic correlation in MZ and DZ twins.

Twin correlation on longitudinal change in each phenotype, i.e. Δphenotype = Pheotype_time2_ –Phenotype_time1_ was estimated by calculating the intra twin pair correlation coefficient (ICC) as $\rho =\frac{\sigma_{s}^{2}}{\sigma_{s}^{2}+\sigma_{e}^{2}}$ with $\sigma_{s}^{2}$ defined as the between pair variance and $\sigma_{e}^{2}$ as the within pair variance in Δphenotype. A higher ICC in MZ twins as compared with DZ twins provides an indication of genetic influence on Δphenotype.

Univariate twin models were fitted to Δphenotype for each of the 12 metabolic traits with sex, age and baseline phenotype level at intake adjusted. For each phenotype, the variance for Δphenotype was decomposed into additive genetic (A), dominant genetic (D), common or shared environmental (C), and unique environmental (E) components. In the model, referred to as ACDE model, C and D cannot be estimated simultaneously in the classical twin study of MZ and DZ twins reared together [32,33]. Two separate models containing the A, C and E components (the ACE model) and the A, D and E components (the ADE model) were fitted with the latter usually preferred when the MZ correlation is more than double the DZ correlation for a given phenotype. Based on the full ACE model, nested models were also fitted by dropping the C (AE model), the A (CE model), or both (E model) components for best model selection. Likewise two nested models (AE and E) were fitted for comparison with the full ADE model. The DE model was excluded because it is biologically implausible considering that the dominant genetic effects alone are not enough to explain the very low DZ correlation when compared with MZ correlation [34]. The likelihood ratio test (LRT) was applied for comparisons on performances between the full models and their nested models. In model comparison, the parsimonious model was preferred when no statistical significance was observed between the two models. Goodness of fit was assessed by calculating the Akaike Information Criterion (AIC) [35]. Robustness of parameter estimates was assessed using bootstrap re-sampling for empirically calculating the 95% confidence intervals (CIs).

In all the analysis, each phenotype value was log transformed to minimize possible skewed phenotype distribution. Phenotype values 3 standard deviations above or below the phenotype mean were set to missing [36]. The mixed effect kinship model was fitted using the free R package *kinship* (http://cran.r-project.org/src/contrib/Archive/kinship/). The calculation of ICC and twin modeling were done by using the free R package *mets* (http://cran.r-project.org/web/packages/mets/index.html).

## Results

### Longitudinal change in metabolic phenotypes

**Fig 1:**
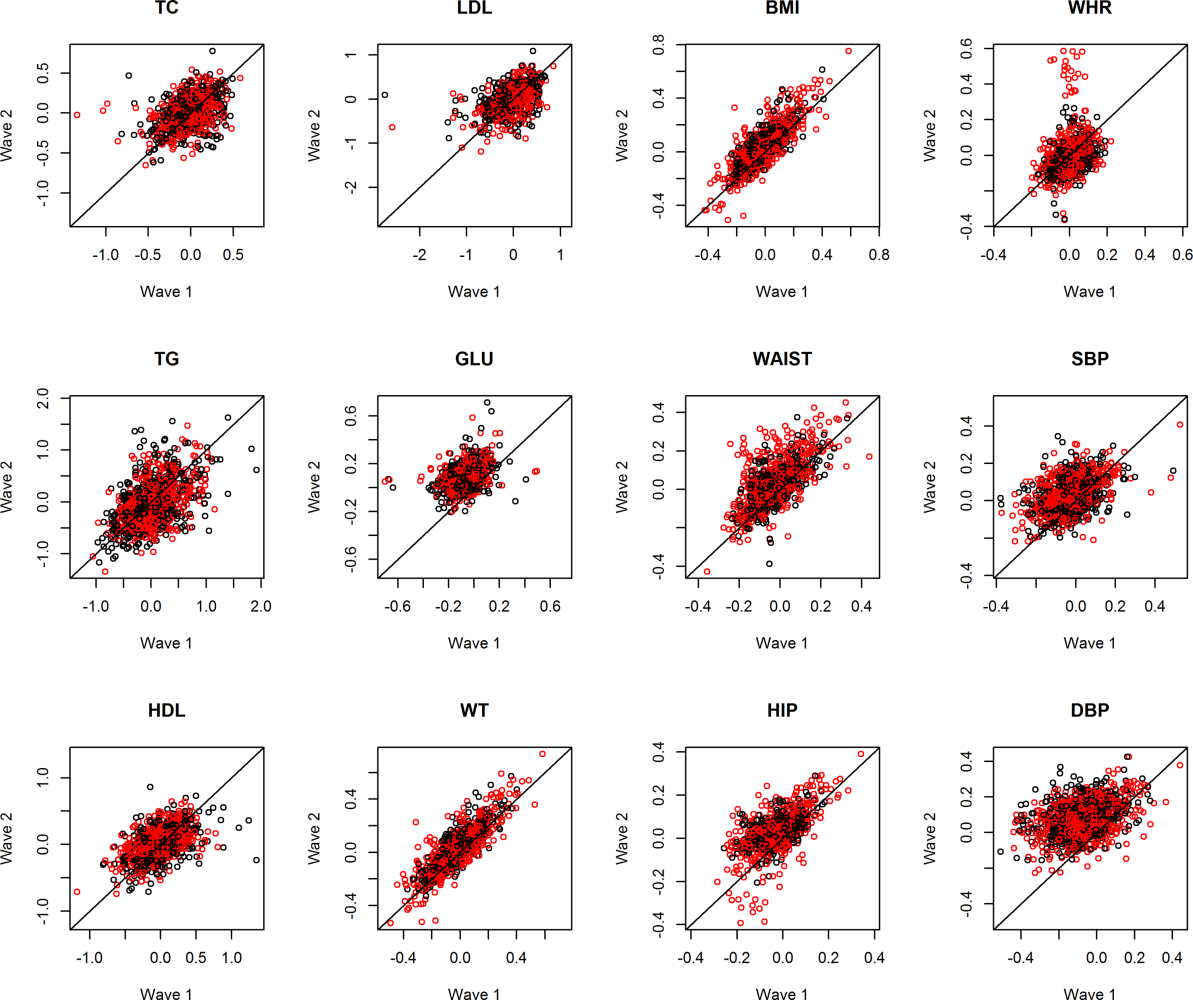
Trend of metabolic phenotypes over time in Danish twins. Scatter plots showing the residuals of phenotype measurement from the mixed effect model at time 1 (horizontal axis) against that at time 2 (vertical axis) for 12 phenotypes in Danish twins (females in red; males in black). TC: total cholesterol; TG: triglycerides; HDL: high density lipoprotein cholesterol; LDL: low density lipoprotein cholesterol; GLU: fasting blood glucose; WT: body weight; BMI: body mass index; WAIST: waist circumference; HIP: hip circumference; WHR: waist-to-hip ratio; SBP: systolic blood pressure; DBP: DP: diastolic blood pressure.

Table 1 shows the basic statistics (mean, 95% CI) for all the metabolic phenotypes at each time point together with the statistical testing on their rate of change with age and sex adjusted. For the Danish twins, statistically significant increases in phenotype values over the follow-up were observed for most phenotypes except for TG and WHR which decreased over time (Table 1). The patterns of longitudinal change in Danish twins are further illustrated in Fig 1 by plotting, for each phenotype, the residuals of phenotype measurement from the mixed effect model at time 1 (horizontal axis) against that at time 2 (vertical axis). Samples with no longitudinal change in the phenotype would fall along the diagonal line from bottom left-hand to top right-hand corner. Patterns that deviate from the diagonal line would indicate increase (above the diagonal) or decrease (below the diagonal) in the phenotype values over the follow-up time. The longitudinal patterns observed in Fig 1 correspond well to the test results from the mixed effects model in Table 1 for the Danish twins. Moreover, Fig 1 reveals no obvious difference in the longitudinal change between females (red dots) and males (black dots) after adjustment for sex in the regression model.

**Table 1:** Basic statistics for baseline (time 1) and follow up (time 2) in Danish and Chinese twins.

	Danish Twins (n = 1004)					Chinese Twins (n = 362)				
Traits	Mean, 1	95% CIs	Mean, 2	95% CIs	P value	Mean, 1	95% CIs	Mean, 2	95% CIs	P value
**TC, mmol/l**	5.36	3.30–8.00	5.48	3.59–7.70	9.47E-07	5.26	3.26–7.60	4.91	2.91–7.01	1.13E-10
**TG, mmol/l**	1.27	0.60–2.90	1.23	0.50–2.90	3.91E-03	1.18	0.38–3.00	1.25	0.37–3.13	3.76E-01
**HDL, mmol/l**	1.52	0.86–2.50	1.55	0.90–2.50	2.90E-05	1.57	0.90–2.25	1.57	0.91–2.59	7.17E-01
**LDL, mmol/l**	3.29	1.50–5.56	3.37	1.70–5.30	4.74E-06	3.10	1.88–4.62	2.68	1.55–4.14	7.24E-33
**GLU, mmol/l**	4.76	3.90–6.00	5.58	4.70–7.00	1.64E-276	4.71	3.50–6.40	5.42	4.12–8.99	3.13E-59
**WT, kg**	73.18	50.30–100.29	76.59	52.60–110.69	1.36E-27	62.70	47.21–87.19	64.03	47.10–88.00	1.83E-06
**BMI, kg/m**^**2**^	24.43	19.03–32.61	25.73	19.42–36.92	5.16E-37	23.89	18.50–31.22	24.45	19.00–31.80	2.98E-08
**WAIST, cm**	83.77	66.00–108.00	88.04	68.00–112.01	2.31E-45	77.32	61.00–97.00	81.94	65.80–106.50	6.29E-18
**HIP, cm**	96.40	81.00–115.00	102.17	86.48–120.00	2.14E-95	96.82	84.15–111.93	96.92	85.00–112.01	3.97E-02
**WHR**	0.87	0.72–1.04	0.86	0.71–1.13	2.62E-02	0.80	0.69–0.92	0.84	0.72–0.97	2.76E-26
**SBP, mmHg**	116.36	93.33–145.33	123.43	101.67–150.00	5.36E-62	118.11	90.00–160.00	125.38	100.00–169.90	1.51E-12
**DBP, mmHg**	68.16	50.67–90.00	79.42	64.50–98.33	1.61E-224	80.36	60.00–103.83	81.55	62.05–109.90	3.20E-02

TC: total cholesterol; TG: triglycerides; HDL: high density lipoprotein cholesterol; LDL: low density lipoprotein cholesterol; GLU: fasting blood glucose; WT: body weight; BMI: body mass index; WAIST: waist circumference; HIP: hip circumference; WHR: waist-to-hip ratio; SBP: systolic blood pressure; DBP: diastolic blood pressure.

**Fig 2:**
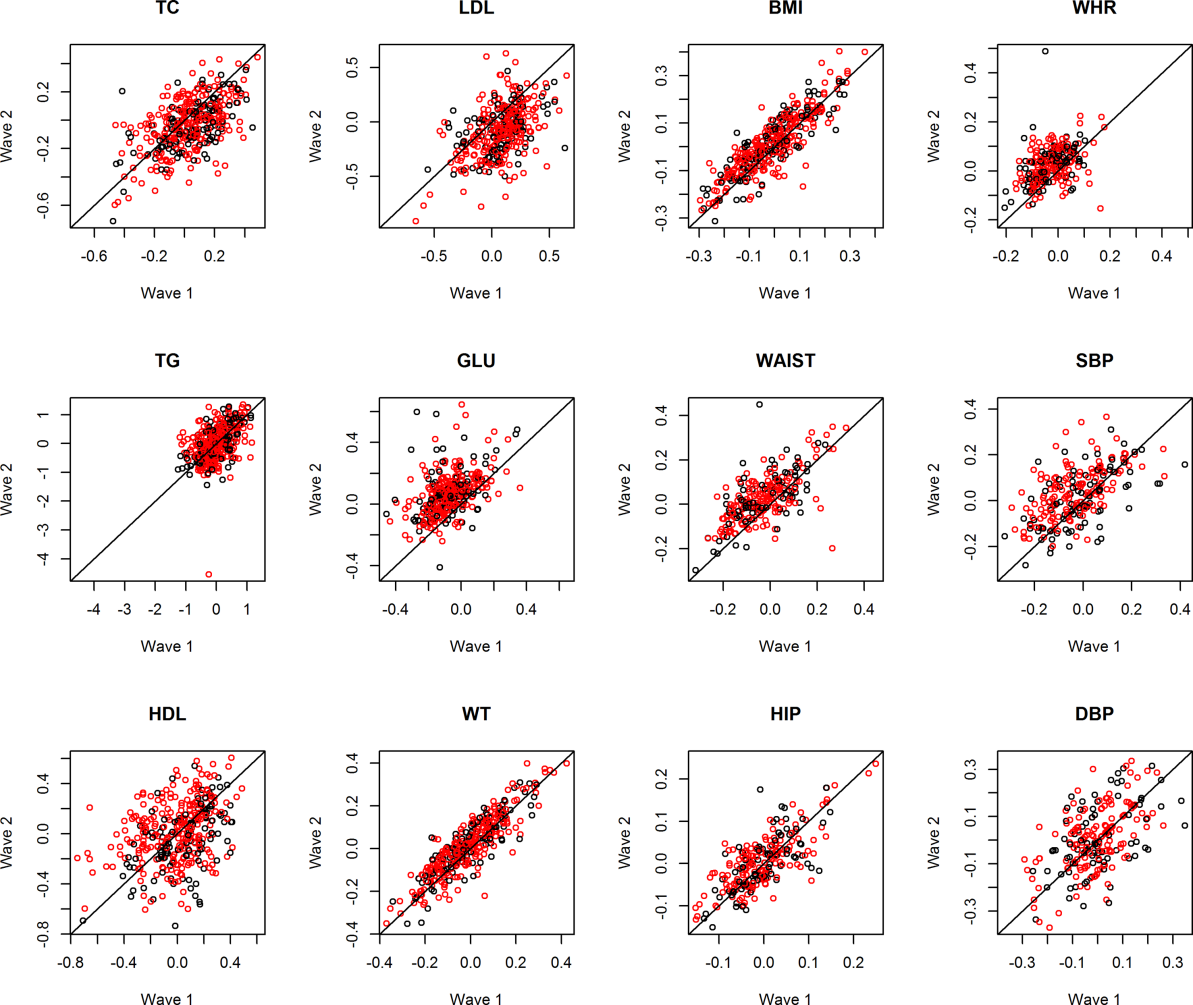
Trend of metabolic phenotypes over time in Chinese twins. Scatter plots showing the residuals of phenotype measurement from the mixed effect model at time 1 (horizontal axis) against that at time 2 (vertical axis) for 12 phenotypes in Chinese twins (females in red; males in black). TC: total cholesterol; TG: triglycerides; HDL: high density lipoprotein cholesterol; LDL: low density lipoprotein cholesterol; GLU: fasting blood glucose; WT: body weight; BMI: body mass index; WAIST: waist circumference; HIP: hip circumference; WHR: waist-to-hip ratio; SBP: systolic blood pressure; DBP: DP: diastolic blood pressure.

For the Chinese twins, the mean value of the 8 phenotypes increased and 2 (TC, LDL) decreased while the mean level of TG and HDL remained unchanged according to their p values (Table 1). Fig 2 is the scatter plot for time points 1 and 2 plotted in the same way as Fig 1. The figure visualizes the results from Table 1 with no obvious sex difference in the longitudinal trends after adjustment for age and sex.

### Twin correlation on longitudinal change of phenotypes

Table 2 presents the ICCs on change in each phenotype (Δphenotype) in MZ and DZ twins for both samples. In the Danish twins, except TG and HIP, all other phenotypes showed higher ICC in MZ than in DZ twins (mostly more than double). In the Chinese twins, no significant differences in ICC between MZ and DZ twins were observed for HDL, DBP and all body mass traits, an indication of limited or lack of genetic control over the longitudinal change in those phenotypes in adult Chinese. Most of the blood biochemical measurements (TC, TG, LDL and GLU) and SBP had higher ICCs in MZ than in DZ Chinese twins. Overall, ICCs on Δphenotype provided evidence of genetic contributions to longitudinal changes in most metabolic phenotypes.

**Table 2:** ICCs for longitudinal change of each phenotype in Danish and Chinese twins.

	Danish Twins				Chinese Twins			
Traits	ICC_MZ_ (n = 452)	95% CIs	ICC_DZ_ (n = 552)	95% CIs	ICC_MZ_ (n = 202)	95% CIs	ICC_DZ_ (n = 160)	95% CIs
**TC**	0.50*	0.38–0.60	0.18	0.06–0.29	0.54	0.37–0.67	0.29	0.06–0.50
**TG**	0.29^Δ^	0.17–0.41	0.26	0.14–0.38	0.58	0.40–0.72	0.37	0.16–0.55
**HDL**	0.47*	0.36–0.57	0.12	0.00–0.24	0.68^Δ^	0.40–0.84	0.63	0.35–0.81
**LDL**	0.51*	0.39–0.61	0.20	0.08–0.31	0.53	0.35–0.68	0.34	0.12–0.53
**GLU**	0.42*	0.29–0.53	0.12	0.00–0.24	0.56	0.38–0.71	0.41	0.19–0.59
**WT**	0.40*	0.27–0.51	0.17	0.06–0.28	0.38^Δ^	0.18–0.54	0.35	0.15–0.53
**BMI**	0.41*	0.29–0.52	0.16	0.04–0.27	0.27^Δ^	0.05–0.46	0.35	0.15–0.51
**WAIST**	0.41*	0.31–0.51	0.13	-0.02–0.27	0.37^Δ^	0.10–0.59	0.46	0.23–0.64
**HIP**	0.44^Δ^	0.33–0.54	0.41	0.29–0.52	0.36^Δ^	0.11–0.57	0.42	0.18–0.62
**WHR**	0.48*	0.37–0.57	0.13	-0.01–0.26	0.49^Δ^	0.22–0.68	0.43	0.21–0.61
**SBP**	0.36	0.22–0.48	0.20	0.08–0.30	0.30*	0.05–0.51	0.10	-0.17–0.36
**DBP**	0.49*	0.37–0.59	0.17	0.06–0.28	0.32^Δ^	0.05–0.55	0.23	-0.01–0.45

*ICC_MZ_>2 times ICC_DZ_

^Δ^ No statistical difference between ICC_MZ_ and ICC_DZ_ with p>0.05.

### Twin modelling on longitudinal change of phenotype

Considering the ICCs for many of the phenotypes in MZ were more than double in DZ twins, both ACE and ADE models were subsequently fitted to each Δphenotype with the model of lower AIC chosen as the full model. Tables 3 and 4 show the parameter estimates in the full model and statistics for the best fitting model for each of the 12 phenotypes in Danish and Chinese twins respectively.

**Table 3:** Full models for longitudinal change of each phenotype in the Danish twins and statistics for best fitting models.

		Parameter estimates						Likelihood Ratio Test	
Traits	Full models	A (95% CIs)	C/D (95% CIs)	E (95% CIs)	AIC	Best models	AIC	*X*^*2*^	*P* value
**TC**	ADE	0.21 (0.00–0.66)	0.29 (0.00–0.77)	0.50 (0.40–0.60)	-786.50	AE	-787.10	1.40	0.24
**TG**	ACE	0.06 (0.00–0.38)	0.23 (0.00–0.49)	0.71 (0.59–0.82)	771.89	CE	770.02	0.12	0.73
**HDL**	ADE	0.02 (0.00–0.48)	0.45 (0.00–0.94)	0.53 (0.43–0.63)	-495.54	AE	-494.54	3.00	0.08
**LDL**	ADE	0.29 (0.00–0.74)	0.22 (0.00–0.70)	0.49 (0.39–0.59)	-121.07	AE	-122.24	0.83	0.36
**GLU**	ADE	0.07 (0.00–0.51)	0.35 (0.00–0.82)	0.58 (0.47–0.69)	-2078.00	AE	-2078.23	1.77	0.18
**WT**	ADE	0.29 (0.00–0.72)	0.10 (0.00–0.56)	0.60 (0.50–0.71)	-2154.63	AE	-2156.46	0.17	0.68
**BMI**	ADE	0.22 (0.00–0.65)	0.19 (0.00–0.65)	0.59 (0.48–0.70)	-2140.59	AE	-2142.01	0.58	0.45
**WAIST**	ADE	0.10 (0.00–0.60)	0.31 (0.00–0.83)	0.59 (0.50–0.68)	-2115.32	AE	-2116.18	1.14	0.29
**HIP**	ACE	0.06 (0.00–0.32)	0.38 (0.17–0.60)	0.56 (0.47–0.66)	-2843.69	CE	-2845.53	0.16	0.69
**WHR**	ADE	0.03 (0.00–0.49)	0.45 (0.00–0.93)	0.52 (0.43–0.61)	-2336.23	AE	-2335.66	2.57	0.11
**SBP**	ACE	0.33 (0.02–0.63)	0.03 (0.00–0.27)	0.64 (0.52–0.76)	-2071.94	AE	-2073.88	0.07	0.80
**DBP**	ADE	0.20 (0.00–0.63)	0.29 (0.00–0.75)	0.51 (0.41–0.61)	-1940.42	AE	-1940.98	1.44	0.23

**Table 4:** Full models for longitudinal change of each phenotype in the Chinese twins and statistics for best fitting models.

		Parameter estimates						Likelihood Ratio Test	
Traits	Full models	A (95% CIs)	C/D (95% CIs)	E (95%CIs)	AIC	Best models	AIC	*X*^*2*^	*P* value
**TC**	ACE	0.49 (0.00–0.97)	0.05 (0.00–0.49)	0.46 (0.33–0.59)	-362.90	AE	-364.85	0.05	0.82
**TG**	ACE	0.42 (0.00–0.84)	0.16 (0.00–0.53)	0.42 (0.29–0.54)	459.65	AE	458.30	0.65	0.42
**HDL**	ACE	0.09 (0.00–0.38)	0.58 (0.33–0.84)	0.32 (0.22–0.42)	-131.95	CE	-133.53	0.42	0.52
**LDL**	ACE	0.38 (0.00–0.84)	0.15 (0.00–0.56)	0.47 (0.33–0.61)	-154.34	AE	-155.86	0.48	0.49
**GLU**	ACE	0.31 (0.00–0.75)	0.25 (0.00–0.63)	0.44 (0.30–0.57)	-495.38	AE	-495.96	1.42	0.23
**WT**	ACE	0.05 (0.00–0.51)	0.33 (0.00–0.70)	0.62 (0.46–0.79)	-965.73	CE	-967.69	0.04	0.84
**BMI**	ACE	0.00 (0.00–0.00)	0.31 (0.18–0.44)	0.69 (0.56–0.82)	-969.89	CE	-971.89	0.00	1.00
**WAIST**	ACE	0.00 (0.00–0.00)	0.42 (0.27–0.57)	0.58 (0.43–0.73)	-546.17	CE	-548.17	0.00	1.00
**HIP**	ACE	0.00 (0.00–0.00)	0.39 (0.24–0.55)	0.61 (0.45–0.76)	-841.78	CE	-843.78	0.00	1.00
**WHR**	ACE	0.11 (0.00–0.62)	0.38 (0.00–0.77)	0.51 (0.31–0.72)	-677.13	CE	-678.97	0.17	0.68
**SBP**	ADE	0.10 (0.00–1.00)	0.20 (0.00–1.00)	0.70 (0.48–0.93)	-437.99	AE	-439.87	0.12	0.73
**DBP**	ACE	0.17 (0.00–0.81)	0.15 (0.00–0.64)	0.68 (0.44–0.92)	-360.47	CE	-362.19	0.27	0.60

For the Danish twins (Table 3), 9 phenotypes were fitted by the ADE model and only three by the ACE model (TG, HIP, SBP) as expected from the ICCs in Table 2. The full models (both ACE and ADE) estimated moderate to high E component in Δphenotype from 0.49 for LDL to 0.71 for TG. In contrast, only low to moderate effects were estimated for the A, C or D components. For most estimates of A, C or D components, the 95% CIs included zero suggesting the need for fitting nested models and for best model selection. In Table 3, the best performance models were also selected for each Δphenotype with the AE model best fitted to 10 phenotypes and the CE model to TG and HIP only. According to AICs in Table 3, all the best fitting models outperformed their full models except for HDL and WHR but none showed statistically significant difference to its full model. As supplementary data, we also provided supporting information (S1 Table) which shows AICs for both full and nested models fitted to Danish (in left hand side) and Chinese (in right hand side) twin data with AICs for the best fitting models marked as bold. Note that the best fitting models again estimated moderate A (from 0.36 for SBP to 0.49 for LDL) and low to moderate C (from 0.28 for TG to 0.43 for HIP) components but, in contrast, high estimates for the E component (from 0.51 for LDL to 0.72 for TG) (Table 5).

**Table 5:** Parameter estimates in best fitting models in the Danish and Chinese twins.

	Danish twins				Chinese twins			
Traits	Best model	A (95% CIs)	C/D (95% CIs)	E (95% CIs)	Best model	A (95% CIs)	C/D (95% CIs)	E (95% CIs)
**TC**	AE	0.48 (0.38–0.57)		0.52 (0.43–0.62)	AE	0.54 (0.42–0.66)		0.46 (0.34–0.58)
**TG**	CE		0.28 (0.19–0.36)	0.72 (0.64–0.81)	AE	0.59 (0.48–0.71)		0.41 (0.29–0.52)
**HDL**	AE	0.44 (0.33–0.54)		0.56 (0.46–0.67)	CE		0.66 (0.57–0.74)	0.34 (0.26–0.43)
**LDL**	AE	0.49 (0.39–0.59)		0.51 (0.41–0.61)	AE	0.54 (0.42–0.67)		0.46 (0.33–0.58)
**GLU**	AE	0.39 (0.28–0.49)		0.61 (0.51–0.72)	AE	0.58 (0.46–0.70)		0.42 (0.30–0.54)
**WT**	AE	0.39 (0.28–0.49)		0.61 (0.51–0.72)	CE		0.36 (0.24–0.49)	0.64 (0.51–0.76)
**BMI**	AE	0.39 (0.29–0.49)		0.61 (0.51–0.71)	CE		0.31 (0.18–0.44)	0.69 (0.56–0.82)
**WAIST**	AE	0.40 (0.30–0.49)		0.60 (0.51–0.70)	CE		0.42 (0.27–0.57)	0.58 (0.43–0.73)
**HIP**	CE		0.43 (0.35–0.50)	0.57 (0.50–0.65)	CE		0.39 (0.24–0.55)	0.61 (0.45–0.76)
**WHR**	AE	0.45 (0.36–0.54)		0.55 (0.46–0.64)	CE		0.45 (0.31–0.60)	0.55 (0.40–0.69)
**SBP**	AE	0.36 (0.26–0.47)		0.64 (0.53–0.74)	AE	0.28 (0.07–0.50)		0.72 (0.50–0.93)
**DBP**	AE	0.47 (0.37–0.56)		0.53 (0.44–0.63)	CE		0.27 (0.10–0.44)	0.73 (0.56–0.90)

For the Chinese twins, 11 phenotypes were fitted by the ACE model with only SBP by ADE model (Table 4). Similar to the Danish twins, all full models estimated moderate to high E component (from 0.32 for HDL to 0.70 for SBP); very low to moderate A, C or D components. As shown in Table 4, all the selected sub-models outperformed their corresponding full models with lower AICs and none displayed significant statistical difference in the goodness of fit as compared to the full models. Likewise, AICs for both the full and the nested models fitted to the Chinese twin data are shown in S1 Table.

Different from the Danish twins that predominantly had the AE model as the best, the various categories of metabolic phenotypes for the Chinese twins were best fitted by different sub-models with the AE model fitted to biochemical measurements (i.e. lipids and glucose except HDL) and the CE model fitted to all body mass traits (Tables 4 and 5). Moreover, the estimated A components for lipids and glucose traits tended to be higher in Chinese twins (from 0.54 for TC and LDL to 0.59 for TG) than in Danish twins (from 0.39 for GLU to 0.49 for LDL) except for HDL (CE model in Chinese twins). Note that, although most of the body mass traits in the two samples were best fitted by different models (AE for Danish and CE for Chinese), one trait, i.e. HIP had consistently the CE model as the best in both Danish and Chinese twins with comparable estimates (Table 5). The blood pressure traits in the Chinese twins were best fitted by the AE model for SBP and CE model for DBP, both with very high E estimates (SBP: 0.72; DBP: 0.73) in comparison with other phenotypes in Chinese twins and also with Danish twins (SBP: 0.64; DBP: 0.53).

## Discussion

By treating the longitudinal change in a phenotype, i.e. Δphenotype as the metrics of interest, we have conducted a longitudinal twin study on multiple metabolic phenotypes in samples from two populations of distinct ethnic background and social environmental circumstances. One important finding in the study is the moderate to high contribution by the unique environment to intra-individual longitudinal change (Δphenotype) for all 12 phenotypes (Table 5). In contrast, the genetic component has only low to moderate contribution to Δphenotype. In summary, the results emphasize the high importance of unique environmental factors in controlling intra-individual variation in metabolic phenotypes over time, both in Danish and in Chinese twins.

In addition to the unique environmental factors, the shared environments were also involved in regulating the longitudinal change of all body mass traits in Chinese twins which is in contrast to the Danish twins. The phenomenon could indicate, in addition to the unique environment, early-life shared environment could also play an important role in determining the individual trajectory of body mass traits in the Chinese adult twins.

In the best fitting models, the genetic estimates to longitudinal changes for lipids (except HDL) and glucose tended to be higher in Chinese than in Danish twins with only a slight overlap in the 95% CIs for GLU (0.58, 95% CI: 0.46–0.70 in Chinese versus 0.39, 95% CI: 0.28–0.49 in Danish twins) but with considerable overlaps for TC and LDL (Table 5). Although the difference lacks strong statistical support for each phenotype considered individually, the same trend of difference (i.e. A for Chinese > A for Danish) in biochemical measurements could reflect interesting population differences in the genetic and environmental control over longitudinal patterns of lipids and glucose. In view of the fact that Chinese twins were sampled from the countryside (the suburban area of Qingdao) where staple food is characterized by high cereal and vegetable content, we assume that the Chinese samples might be more restricted in their dietary pattern being much more plant based than the Danish twins who had more sufficient food supply and in general have a dietary pattern that includes high intakes of animal-based food [37–38]. As a result, the difference in dietary habits between the two samples could lead to low unique environmental and high genetic components in the variation of Δphenotype for blood lipids and glucose in the Chinese twins, while high unique environmental and low genetic components in the Danish twins. Future cross-population studies should help to validate our hypothesis.

Among the lipid phenotypes, no genetic component was estimated in the best fitting models (i.e. the CE model) for ΔTG in Danish twins and for ΔHDL in the Chinese twins. The absence of genetic control over ΔTG is consistent with Friedlander et al [39] who reported no genetic influence on the change in TG over a 10-year follow-up in an adult cohort of American twins. In another longitudinal study conducted in adult Caucasian twins, Goode et al. [40] reported no significant proportion of genetic contribution to the variation in age-related change of blood lipids. Different from the results in adult twins, Middelberg et al. [41] and Zhang et al. [25] estimated significant genetic component in age-related change on the level of blood lipids in adolescent Caucasian and Chinese twins respectively. Comparing the results for adolescent and adult twins, one could conclude that the genes are important in regulating the developmental changes of blood lipids in adolescent twins in both Eastern and Western populations while in adult twins, the genetic effects on long-term change for some lipids (here TG in Danish twins and HDL in Chinese twins) could have been weakened and perhaps with population-specific patterns.

Different from the lipids and glucose phenotypes, longitudinal change in blood pressure was highly attributable to unique environment in Chinese twins (0.72 for SBP, 95% CI: 0.50–0.93; 0.73 for DBP, 95% CI: 0.56–0.90). The estimates of E components for the change of blood pressures in Danish twins (0.64 for SBP, 95% CI: 0.53–0.74; 0.53 for DBP, 95% CI: 0.44–0.63) tended to be lower than that for the Chinese twins although their 95% CIs overlapped. On the other hand, the Danish twins had moderate genetic influence on change in blood pressure (0.36, 95% CI: 0.26–0.47 for SBP; 0.47, 95%CI: 0.37–0.56 for DBP) which is in contrast to the lower or no genetic control in the Chinese twins (0.28 for SBP, 95% CI: 0.07–0.50; 0 for DBP). Although the different patterns could be ascribed to the different ethnic (genetic) backgrounds, we emphasize the importance of salt consumption in China especially in the rural areas. According to a global epidemiological study, China was on the top rank in dietary salt intake [42] and the intake level changed with age [43]. We think that the high contribution by unique environment to change in blood pressure can be, at least, partly explained by the high level of salt intake in China considering the significantly positive association of salt intake with blood pressure [42]. If this was the case, the high salt intake affects not only the variation in the level [44–47] but also in the variation in the rate of change of blood pressure in the Chinese population.

This longitudinal twin analysis was based on intra-pair correlation (ICC) on Δphenotype over time, which did not necessarily imply significant longitudinal change at the mean level of the phenotype. For example, the mean level of HDL in the Chinese twins had no significant change over time (p = 0.72) (Table 1) but high ICCs on ΔHDL were estimated for both MZ (0.68, 95% CI: 0.40–0.84) and DZ (0.63, 95% CI: 0.35–0.81) twins (Table 2) which led to a best fitting CE model (Table 5). In another example, the mean level for TG had no significant change over the follow-up period in the Chinese twins (p = 0.38) (Table 1). However, the estimated ICCs for TG were higher in MZ (0.58, 95% CI: 0.40–0.72) than in DZ (0.37, 95% CI: 0.16–0.55) twins (Table 2) suggesting genetic involvement in the intra-individual change over time. This was confirmed by the best fitted AE model for TG (Table 4) with A component counting for 59% of the total variance in ΔTG (Table 5). If our results on TG are validated, we can assume that there could have been genetic polymorphisms inherited by different twin pairs that up- or down-regulated TG with comparable effect size in each direction and eventually resulted in no change at the overall mean level of TG. The two examples demonstrate the need to differentiate the genetic and environmental control over intra-individual longitudinal change from that over the level of a phenotype. By applying the growth curve model to multi-wave measurements on BMI, Hjelmborg et al. [22] were able to show that the genetic variants for longitudinal change in BMI were likely to be different from those affecting the level of BMI. The estimated genetic regulation of intra-individual phenotype variation could help to guide genetic association studies to look specifically for genes that influence the rate of change in multiple metabolic phenotypes.

It is necessary to point out the limitations of our comparative study. First, the Danish and Chinese twins were followed up for different length of time which could possibly result in different degrees of accumulation for the random environmental effects on Δphenotype. Although the estimated E components in Table 5 do not seem to support the speculation, we cannot rule out the existence of differential accumulation effects in the two samples. Second, in both samples, phenotypes were measured at only two time points. Because of that, it was impossible to fit the growth curve model and thus it was unable to estimate the genetic and environmental effects in the correlation between rate of change and baseline level of the phenotypes. Third, the small sample size of Chinese twins could be responsible for the insignificant results on the mean value of longitudinal change in TG and HDL and likewise higher uncertainty in the parameter estimates for the twin models.

## Conclusion

Our study emphasizes the major role of individual unique environment in controlling the intra-individual variation over time in metabolic phenotypes in both Danish and Chinese twins, and meanwhile, indicates differential patterns of genetic and common environmental regulations on the long-term intra-individual change in different clusters of metabolic phenotypes in the two samples.

## Supporting Information

S1 Table AICs for the full and nested models for Danish and Chinese twins. The table presents AICs for all models fitted (both full and nested models) with the AICs for the best fitting models marked as bold.
(DOCX)
