# Supplementary material for: Genetic and Environmental Regulation on Longitudinal Change of Metabolic Phenotypes in Danish and Chinese Adult Twins
Source: PLoS One. 2016 Feb 10;11(2):e0148396. doi: 10.1371/journal.pone.0148396 (PMC4749287; doi:10.1371/journal.pone.0148396)
Supplement: S1 Table — The table presents AICs for all models fitted (both full and nested models) with the AICs for the best fitting models marked as bold. (DOCX) [file pone.0148396.s001.docx]

| **S1 Table. AICs of full and nested models for both Danish and Chinese twins (best models marked bold)** | | | | | | | | | | | |
| --- | --- | --- | --- | --- | --- | --- | --- | --- | --- | --- | --- |
|  | **Danish twins** | | | | |  | **Chinese twins** | | | | |
| **Traits** | **ACE** | **ADE** | **AE** | **CE** | **E** |  | **ACE** | **ADE** | **AE** | **CE** | **E** |
| **TC** | -785.10 | -786.50 | **-787.10** | -771.16 | -724.15 |  | -362.90 | -362.85 | **-364.85** | -360.61 | -323.48 |
| **TG** | 771.89 | 774.91 | 772.91 | **770.02** | 805.72 |  | 459.65 | 460.30 | **458.30** | 461.75 | 505.00 |
| **HDL** | -492.54 | -495.54 | **-494.54*** | -479.39 | -442.65 |  | -131.95 | -120.65 | -122.65 | **-133.53** | -36.94 |
| **LDL** | -120.24 | -121.07 | **-122.24** | -107.56 | -60.81 |  | -154.34 | -153.86 | **-155.86** | -153.59 | -118.07 |
| **GLU** | -2076.23 | -2078.00 | **-2078.23** | -2067.53 | -2041.06 |  | -495.38 | -493.96 | **-495.96** | -495.29 | -450.59 |
| **WT** | -2154.46 | -2154.63 | **-2156.46** | -2148.99 | -2115.78 |  | -965.73 | -963.34 | -965.34 | **-967.69** | -944.25 |
| **BMI** | -2140.01 | -2140.59 | **-2142.01** | -2123.80 | -2100.53 |  | -969.89 | -966.27 | -968.27 | **-971.89** | -955.98 |
| **WAIST** | -2114.18 | -2115.32 | **-2116.18** | -2105.41 | -2061.55 |  | -546.17 | -541.23 | -543.23 | **-548.17** | -527.62 |
| **HIP** | -2843.69 | -2834.04 | -2836.04 | **-2845.53** | -2751.53 |  | -841.78 | -838.26 | -840.26 | **-843.78** | -826.17 |
| **WHR** | -2333.66 | -2336.23 | **-2335.66*** | -2319.11 | -2268.89 |  | -677.13 | -674.31 | -676.31 | **-678.97** | -654.33 |
| **SBP** | -2071.94 | -2071.88 | **-2073.88** | -2070.23 | -2040.83 |  | -437.87 | -437.99 | **-439.87** | -438.68 | -436.13 |
| **DBP** | -1938.98 | -1940.42 | **-1940.98** | -1925.96 | -1884.01 |  | -360.47 | -360.16 | -362.16 | **-362.19** | -355.80 |
| *****Selected as best fitting model because no significant difference from the full model with p>0.05.  TC: total cholesterol; TG: triglycerides; HDL: high density lipoprotein cholesterol; LDL: low density lipoprotein cholesterol; GLU: fasting blood glucose; WT: body weight; BMI: body mass index; WAIST: waist circumference; HIP: hip circumference; WHR: waist-to-hip ratio; SBP: systolic blood pressure; DBP: diastolic blood pressure. | | | | | | | | | | | |
